# Role of Dietary Flavonoid Compounds in Driving Patterns of Microbial Community Assembly

**DOI:** 10.1128/mBio.01205-19

**Published:** 2019-09-24

**Authors:** Kerry L. Ivey, Andrew T. Chan, Jacques Izard, Aedin Cassidy, Geraint B. Rogers, Eric B. Rimm

**Affiliations:** a Department of Nutrition, Harvard T. H. Chan School of Public Health, Boston, Massachusetts, USA; b Infection and Immunity Theme, South Australian Health and Medical Research Institute, Adelaide, South Australia, Australia; c Clinical and Translational Epidemiology Unit, Massachusetts General Hospital, Boston, Massachusetts, USA; d Harvard Medical School, Boston, Massachusetts, USA; e Broad Institute of Massachusetts Institute of Technology and Harvard, Cambridge, Massachusetts, USA; f Department of Infectious Diseases, Harvard T. H. Chan School of Public Health, Boston, Massachusetts, USA; g Food Science and Technology Department, University of Nebraska, Lincoln, Nebraska, USA; h School of Biological Sciences, University of Nebraska, Lincoln, Nebraska, USA; i Fred and Pamela Buffet Cancer Center, Lincoln, Nebraska, USA; j Institute for Global Food Security, Queen's University Belfast, Belfast, Northern Ireland; k College of Medicine and Public Health, Flinders University, Adelaide, South Australia, Australia; l Department of Medicine, Brigham and Women’s Hospital, Channing Division of Network Medicine, Boston, Massachusetts, USA; Beijing Tuberculosis and Thoracic Tumor Research Institute, Beijing Chest Hospital, Capital Medical Unversity; University of Hawaii at Manoa

**Keywords:** diet, flavonoid, microbiome

## Abstract

Flavonoids are a group of polyphenolic dietary compounds found in many different plant-based foods. There is increasing evidence that higher flavonoid intake may be causally linked to a reduced risk of cardiovascular disease and other chronic diseases. The bioactivity and bioavailability of many dietary flavonoids can be influenced by gastrointestinal microbiome metabolism. However, the role that habitual flavonoid intake plays in shaping the human gut microbiome is poorly understood. We describe an application of an ecosystem-based analytic approach to nutritional, microbiome, and questionnaire data from a cohort of more than 240 generally healthy adult males to assess the role of dietary flavonoid compounds in driving patterns of microbial community assembly. We identified six subclass-specific microbial communities (SMCs) uniquely and independently associated with intakes of the six flavonoid subclasses. Eggerthela lenta was positively associated with intakes of flavonol and flavanone, and Adlercreutzia equolifaciens was positively associated with intakes of flavonols and flavanol monomers. In contrast, for nearly all flavonoid subclasses, Flavonifractor plautii was inversely associated with subclass consumption. Consuming tea at least once per week explained 10.4% of the total variance in assembly of the 20 species comprising the flavanol monomer SMC. The novel methodology employed, necessitated by multidimensional microbiome data that consist of nonindependent features that exhibit a wide range of distributions and mean values, addresses a major challenge in our ability to understand associations of the microbiome in a wide range of clinical and epidemiologic settings.

## Abstract (precis)

Dietary flavonoids, which have been implicated in lowering chronic disease risk and improving blood pressure, represent a diverse group of polyphenolic compounds found in many commonly consumed foods such as tea, red wine, apples, and berries. The bioactivity and bioavailability of more dietary flavonoids can be influenced by gastrointestinal microbiome metabolism. With demonstrated prebiotic and antimicrobial effects in *in vitro* and in animal models, it is surprising that there are not many human studies investigating the role dietary flavonoids play in shaping the gastrointestinal microbiome. Our analysis revealed patterns of community assembly that uniquely and independently characterize an individual’s exposure to various flavonoid compounds. Furthermore, this study confirmed, independent from effects of other dietary and lifestyle factors included in the multivariate-adjusted model, that flavonoid intake is associated with microbial community assembly.

## INTRODUCTION

Flavonoids are a group of structurally diverse, water-soluble, polyphenolic, plant-derived compounds found in nutritionally diverse foods, including apples, berries, tea, citrus, and red wine (1–3). There is increasing evidence that higher flavonoid intake may be causally linked to a reduced risk of cardiovascular disease and other chronic diseases. These benefits appear to result from improvements in nitric oxide homeostasis and endothelial function and reductions in platelet aggregation and oxidative stress (4–19). The ubiquity of flavonoids and their metabolites in the food supply worldwide makes them an important candidate for population-level prevention of chronic disease. It has long speculated that microbial metabolism might be a major contributor to the overall metabolism of dietary flavonoids (20–26).

The mechanisms underpinning microbial metabolism of dietary flavonoids and their absorption by the host are well defined (26). However, the role that habitual flavonoid intake plays in shaping the human gut microbiome, and by extension, on microbiome-mediated nutritional and physiological impacts, is poorly understood. Elucidating the interplay between dietary flavonoids and gut microbiology is essential if their role in mitigating risks for pathologies, including cardiovascular disease, diabetes, cognitive function, and cancer (4–10, 27, 28), is to be exploited effectively.

We aimed to identify the relationships between the patterns of consumption of specific flavonoid subclasses and the composition of the human gut microbiome. Specifically, we set out to test the hypothesis that there is significant divergence in the gut microbiome characteristics of habitual high and low flavonoid consumers, including in the representation of flavonoid-metabolizing taxa.

The potential complexity of interactions between dietary flavonoids and the human gut microbiota presents a major analytical challenge when testing this hypothesis. Derivations of the conserved 2-phenylchroman nucleus flavonoid structure give rise to more than 4,000 different flavonoid compounds, grouped into six different flavonoid subclasses, and each with unique chemical and physiological properties (2). When combined with the phylogenetic and functional diversity of the human gut microbiota, the potential complexity of interactions involving gut microbes, flavonoids, and their derivative metabolites, is vast. This complexity is compounded by the capacity of flavonoids to promote or suppress bacterial growth (23–25, 29–31).

Multidimensional microbiome data sets are characterized by variables that are neither biologically nor numerically independent and that exhibit a wide range of distributions and mean values. Analyses of diet-microbiome associations are based on the biological hypothesis that dietary intake has a profound influence on microbiome composition (32, 33). In order for statistical models to reflect this hypothesis, dietary variables need to represent the independent variable, and the numerous microbiome variables need to represent the dependent variable matrix. In nutritional epidemiology, diet association studies are plagued by a multitude of potential diet, lifestyle, and environmental factors that may influence observed population-level associations (34). To combine the many nuances associated with nutritional epidemiology analyses with the biological complexities underpinning ecological analyses, we used novel implementations of statistical methods developed and validated previously (35), combined with structural equation modeling and regression-based analyses, to identify and interrogate subclass-specific microbial communities (SMCs).

We describe an application of this approach to nutritional, microbiome, and validated questionnaire data from a cohort of generally healthy adult males to describe the role of dietary flavonoid compounds (and their main dietary sources in the habitual diet) in driving patterns of microbial community assembly.

## RESULTS AND DISCUSSION

### Cohort characteristics.

The characteristics of the study population are presented in Table 1. The mean ± standard deviation total-flavonoid intake was 460 ± 303 mg/day, with the highest tertile of high total-flavonoid consumers having more than threefold-greater mean total-flavonoid intake than low total-flavonoid consumers. This biologically substantial difference of 570 mg/day equates to nearly three cups of black tea, nine medium sized oranges, or nearly five glasses of red wine (36, 37). High total-flavonoid consumers were more likely to have their samples collected in winter and be of healthy weight (as defined as having a body mass index of <25 kg/m^2^). The cohort consisted of only four current smokers, two from the low total-flavonoid consuming group, and two from the moderate consumers. There was no difference in fecal microbial alpha diversity across the different levels of total-flavonoid intake.

**TABLE 1:** Baseline cohort characteristics stratified by total-flavonoid consumption tertiles

Characteristic	Parameter value^*a*^		
	Low flavonoid intake (<300 mg/day)	Moderate flavonoid intake (300 to ≤505 mg/day)	High flavonoid intake (>505 mg/day)
No. of participants	83	81	83
Total flavonoid intake (mg/day)	206 ± 64	394 ± 61	779 ± 309
Alpha diversity (Shannon H’ index)	3.9 ± 0.2	3.9 ± 0.3	3.9 ± 0.3

Sample details and demographics			
Bristol score^*b*^			
Type 1-2 (%)	17	12	13
Type 3-4 (%)	69	72	69
Type 5-7 (%)	14	16	18
Season of sample collection			
Summer (%)	11	14	10
Autumn (%)	11	17	18
Winter (%)	37	25	49
Spring (%)	41	44	23
Sample collected in the morning (%)	82	83	86
Geographical location			
West (%)	30	28	22
Midwest (%)	22	26	24
South (%)	31	30	30
Northeast (%)	17	16	24

Characteristics and medications			
Age (yr)	71 ± 4	71 ± 4	71 ± 5
Physical activity (METs^*c*^)	112 ± 62	113 ± 51	134 ± 56
Body mass index^*d*^			
Normal wt (%)	49	47	59
Overweight (%)	36	44	31
Obese (%)	14	9	10
Antibiotics (%)^*e*^	27	30	23
Acid-lowering medications (%)^*f*^	24	17	18

Dietary intake (mean ± SD)			
Caloric intake (kcal/day)	1895 ± 513	2151 ± 590	2464 ± 562
Protein intake (g/day)^*g*^	84 ± 14	83 ± 15	81 ± 14
Fat intake (g/day)^*g*^	76 ± 13	75 ± 13	72 ± 14
Saturated fat (g/day)^*g*^	24 ± 5	22 ± 5	21 ± 6
Trans fat (g/day)^*g*^	2.2 ± 0.6	2.0 ± 0.6	1.8 ± 0.5
Monounsaturated fat (g/day)^*g*^	29 ± 6	30 ± 8	28 ± 7
Polyunsaturated fat (g/day)^*g*^	16 ± 4	16 ± 4	17 ± 5
Carbohydrate intake (g/day)^*g*^	224 ± 33	229 ± 36	241 ± 38
Alcohol intake (g/day)^*g*^	16 ± 18	18 ± 18	20 ± 20
Fiber intake (g/day)^*g*^	23 ± 6	25 ± 6	28 ± 7
Yogurt intake (servings/wk)	1.6 ± 2.1	2.5 ± 2.5	3.3 ± 3.5

a Results are percentages or means ± standard deviations (SDs), where appropriate. A total of 247 participants were examined in this study.

b As defined by the Bristol stool chart.

c METs, metabolic equivalents.

d Body mass index (BMI) cutoffs: normal weight (<25 mg/kg^2^), overweight (25 to <30 mg/kg^2^), obese (≥30 mg/kg^2^).

e Use of antibiotics reported in the preceding 12 months.

f Use of proton pump inhibitors and/or H_2_ receptor antagonists reported in the preceding 2 months.

g Values are energy adjusted.

Similar to non-U.S. countries (38–40), the flavanol polymer and monomer classes made the greatest contributions to total-flavonoid intake in this cohort, contributing 62% and 12%, respectively (Table 2). Despite the fact that the study population was a U.S. population, the foods contributing most to flavonoid intake are common features of many dietary patterns (41, 42).

**TABLE 2:** Structure and intake of flavonoid subclasses and the major whole-food contributors in this cohort

Flavonoid subclass	Subclass intake (mg/day)^*a*^	Characterizing structure	Major food source	Contribution to subclass intake (%)
Flavonols	26 ± 14	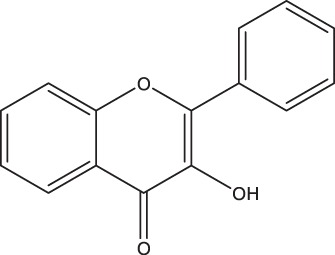	Onion	23.0
			Tea	12.7
			Apple	9.4
Flavanol monomers	57 ± 55	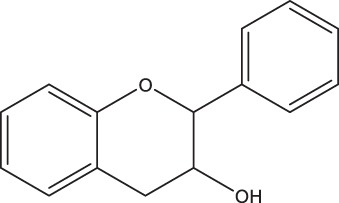	Tea	41.5
			Blueberry	16.6
			Red wine	10.3
Flavanol polymers	283 ± 207	Polymeric compounds	Tea	28.1
			Apple	15.8
			Blueberry	11.4
Flavanones	38 ± 36	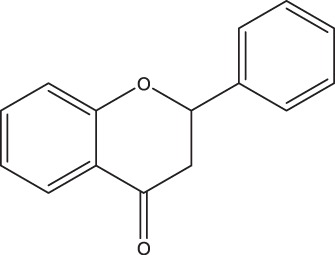	Oranges^*b*^	83.5
			Grapefruit^*b*^	8.3
			Red wine	3.8
Flavones	3.7 ± 2.9	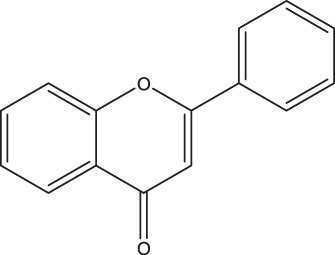	Oranges^*b*^	29.6
			Red wine	23.2
			Vegetable juice	18.6
Anthocyanidins	52 ± 46	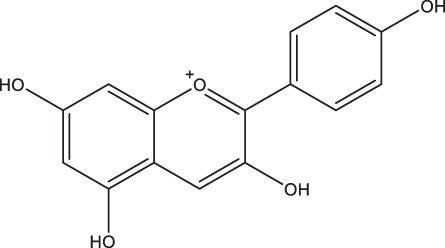	Blueberry	63.1
			Strawberry	12.1
			Apple	9.2

a Results are means ± standard deviations (SDs). There were a total of 247 participants in this study.

b Includes both juice and the whole fruit.

### Composition of the subclass-specific microbial communities.

We first sought to identify subclass-specific communities (SMCs) in order to summarize the species that were differentially more, and less, prevalent in high flavonoid subclass consumers. For all flavonoid subclasses, the greatest degree of discrimination between low and high subclass consumers was observed along eigenvector 1 of the canonical discriminant models, and thus, the eigenvector 1 canonical discriminant coefficients were used to determine which microorganisms comprise SMC scores. This ecosystem-based analysis identified six communities of bacteria, one for each flavonoid subclass.

The *Lachnospiraceae* (NCBI taxonomy identifier NCBI:txid186803), *Prevotellaceae* (NCBI:txid171552), *Coriobacteriaceae* (NCBI:txid84107), and *Bacteroidaceae* (NCBI:txid815) families were the most bacterial common families to be identified, representing 20%, 10%, 10%, and 9%, respectively, of inclusions into individual SMC scores.

Members of the *Bacteriodaceae* family made predominantly inverse contributions to subclass-specific microbiome communities. Antimicrobial effects of flavonoids on *Bacteriodaceae* have been observed in chicks and broilers (43, 44), and tea polyphenols have been reported to reduce the abundance of *Bacteriodaceae* in the fecal samples of swine (45). *In vitro*, tea flavonoids and their metabolites have been shown to inhibit the growth of bacteria belonging to the *Bacteroides* genus (46). Together, these results suggest that this broad antimicrobial effect of flavonoids on *Bacteriodaceae* is less likely to be linked to specific enzymes, and more likely to occur as a result of broadly conserved structural features in the *Bacteriodaceae* family.

Of the 73 species identified for inclusion into the SMC scores, 56% (*n* = 41) represented species that were unique to just one flavonoid subclass, with anthocyanidins having the most nonshared species (Table 3). Anthocyanidins are differentiated from all other flavonoid subclasses by being the only flavonoid subclass to exist naturally in ionic form.

**TABLE 3:**
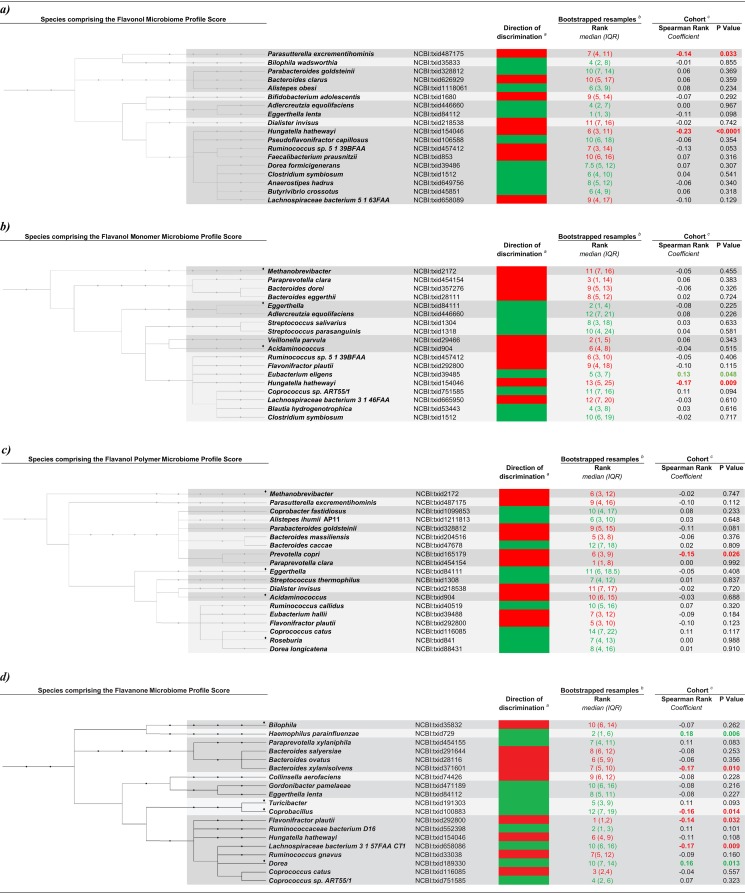

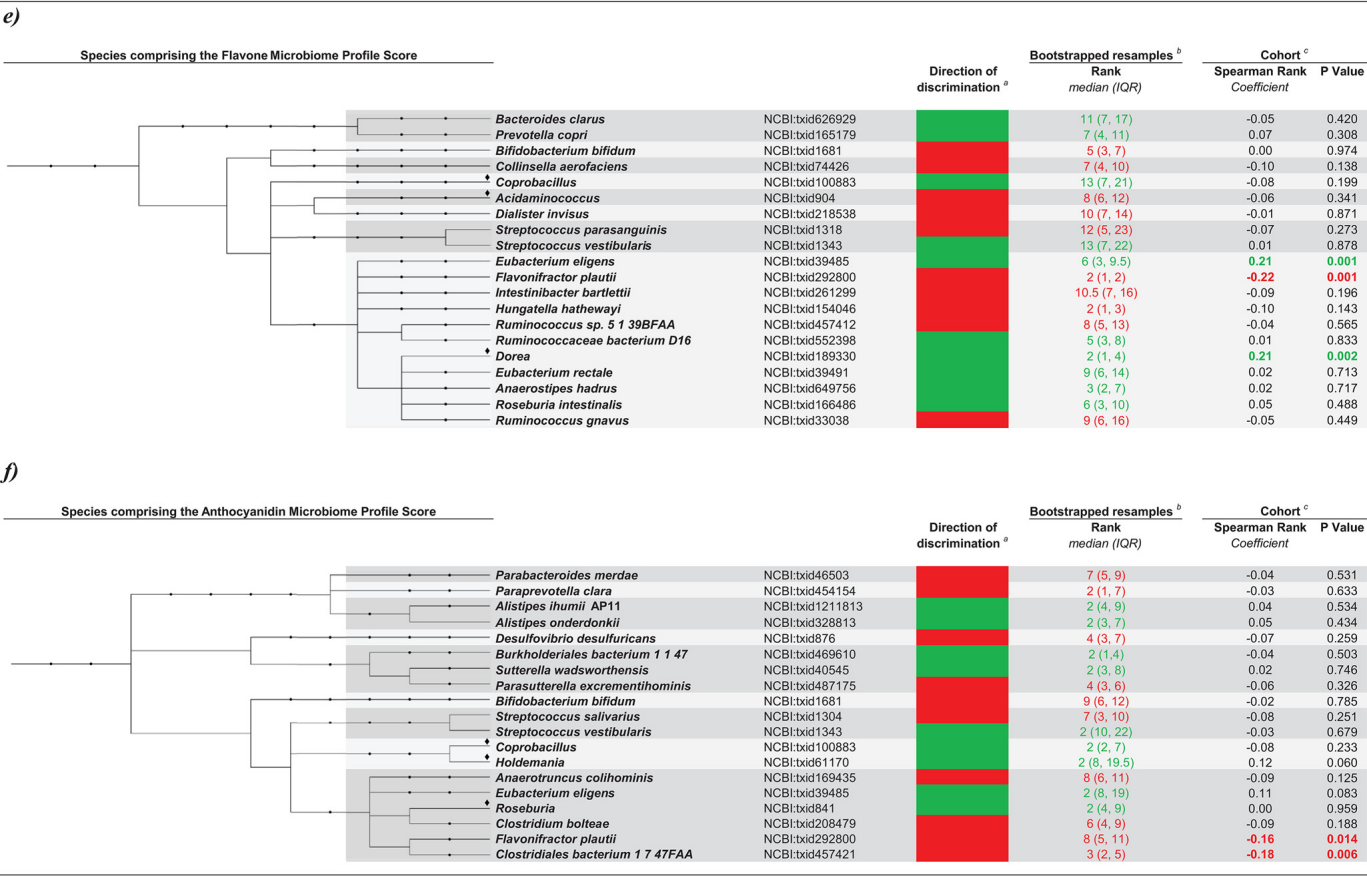
Species composition of subclass-specific microbiome profile scores and their relation with subclass intake^*a*^

a Panel a shows species composition of flavonol microbiome profile scores and their relationship with flavonol intake. Unclassified groups of *Bilophila* (NCBI:txid35832) and *Eggerthella* (NCBI:txid84111) are also included in this profile as inverse discriminators. Panel b shows species composition of flavanol monomer microbiome profile scores and their relationship with flavanol monomer intake. Unclassified groups of *Veillonella* (NCBI:txid29465) and *Paraprevotella* (NCBI:txid577309) are also included in this profile as positive discriminators. Panel c shows species composition of flavanol polymer microbiome profile scores and their relation with flavanol polymer intake. An unclassified group of *Paraprevotella* (NCBI:txid577309) is also included in this profile as a positive discriminator. Panel d shows species composition of flavanone microbiome profile scores and their relationship with flavanone intake. An unclassified group of *Paraprevotella* (NCBI:txid577309) is also included in this profile as an inverse discriminator. Panel e shows species composition of flavone microbiome profile scores and their relationship with flavone intake. Panel f shows species composition of anthocyanidin microbiome profile scores and their relationship with anthocyanidin intake. An unclassified group of *Paraprevotella* (NCBI:txid577309) is also included in this profile as a positive discriminator. For all panels, the subclass-specific Bonferroni corrected level of significance is *P* value of <0.0025. For the Direction of discrimination column, footnote *a* indicates that red species represent species in the top ten of inverse subclass-specific discriminatory rankings and green species represent species in the top ten of positive discriminatory rankings for each given subclass and that only species in terminal nodes are displayed. For the Bootstrapped resamples column, footnote *b* indicates the direction-specific median (interquartile range [IQR]) ranking of the canonical coefficient from 500 bootstrap resamples. For the Cohort column, footnote *c* indicates that results are partial Spearman rank correlation coefficients, controlling for the following variables: age at time of fecal sample collection; energy expended in physical activity; body mass index; and intakes of yogurt, calories, protein, saturated fat, trans fat, carbohydrate and fiber. *n* = 247. Black diamonds indicate unclassified species within the stated genus.

The species identified in each SMC are phylogenetically diverse (Table 3). This is to be expected given that the compounds comprising each flavonoid subclass also have diverse structures, and there are numerous factors dictating how compounds elicit antimicrobial effects. In agreement with our data, a comprehensive review of the BRENDA (47) database (see Table S1 in the supplemental material) reveals that there is great phylogenetic diversity in the microorganisms reported as being capable of flavonoid metabolism. As is the case for flavonoid-microbe literature, the BRENDA database does not provide a comprehensive summary of all bacteria involved in flavonoid metabolism, but rather, it is useful in indicating the diversity of ways in which bacteria metabolize flavonoids. For example, we identified Bifidobacterium adolescentis (NCBI:txid1680) was inversely discriminatory for flavonol intake (Table 3, panel a). The BRENDA database identifies B. adolescentis as a flavanol monomer metabolizer by acting upon sucrose and (+)-catechin to produce d-fructose, (+)-catechin 3′-*O*-alpha-d-glucoside, and (+)-catechin 3′,5-*O*-alpha-d-diglucoside. *B. adolescentis* catalyzes a similar reaction involving (−)-epicatechin to produce (−)-epicatechin 3′-*O*-alpha-d-glucoside, (−)-epicatechin 5-*O*-alpha-d-glucoside, and (−)-epicatechin 3′,5-*O*-alpha-d-diglucoside (47). The precise mechanism by which flavonol compounds interact with *B. adolescentis*, both enzymatically and structurally, remains unclear.

10.1128/mBio.01205-19.2 TABLE S1 Flavonoid-metabolizing bacterial species identified in the BRENDA database, with the associated cosubstrate (blue panel). Download Table S1, DOCX file, 0.2 MB. Copyright © 2019 Ivey et al. 2019 Ivey et al. This content is distributed under the terms of the Creative Commons Attribution 4.0 International license.

Eggerthella lenta (NCBI:txid84112) was positively associated with higher intakes of both flavonols and flavanones. Red wine is a major contributor to flavanone intake in this cohort and is also a good source of dietary flavonols. A randomized controlled trial, also in healthy adult men, found that supplementation with red wine increased the fecal content of E. lenta compared to placebo, independent of its alcohol content, and was positively associated with the concentration of red wine metabolites in the urine (48).

Eggerthella lenta is a flavonoid-metabolizing species (49, 50), strains of which are capable of reductively cleaving the heterocyclic C-ring that is characteristic of flavonoid compounds (51). For example, *E. lenta* rK3 cleaved the heterocyclic C-ring of both (−)-epicatechin and (+)-catechin, giving rise to 1-(3,4-dihydroxy-phenyl)-3-(2,4,6-trihydroxyphenyl)propan-2-ol. This effect does not appear to be strain specific, but rather a feature of *E. lenta* in general (52). Furthermore, *E. lenta* JCM 9979 has been shown to convert (−)-epigallocatechin into 1-(3,4,5-trihydroxyphenyl)-3-(2,4,6-trihydroxyphenyl) propan-2-ol (53). Even though the literature shows mechanisms for *E. lenta* in the context of flavanol monomers, it is reasonable to draw the conclusion that this species is capable of metabolizing flavonoid compounds more broadly.

Conversely, we have direct evidence for other identified species where the literature has investigated the exact subclass species pairs we observed. For example, we found that Adlercreutzia equolifaciens (NCBI:txid 446660) was a positive discriminator for both flavonol and flavanol monomer intake, meaning that A. equolifaciens is present in greater abundance in high, compared to low, consumers of flavonols and flavanol monomers. *A. equolifaciens* has been shown to metabolize flavanol monomers (49) by catalyzing cleavage of the central C-ring, and then dehydroxylated the 4’ carbon of the resultant metabolite’s B-ring (50, 53–55). Although traditionally thought of as an isoflavone-metabolizing bacterium, more-recent data have demonstrated the capacity of *A. equolifaciens* to metabolize nonisoflavone compounds (50), further reinforcing the concept that activities of flavonoid-metabolizing bacteria are often not constrained to one single flavonoid subclass.

Inverse discriminators are considered to be species that, when considered a part of a community, are present in smaller relative abundance in high, compared to low, flavonoid subclass consumers. Flavonifractor plautii (NCBI:txid292800) was an inverse discriminator in the microbiome communities for five of the six flavonoid subclasses: flavanol monomers, flavanol polymers, flavanones, flavones, and anthocyanidins. F. plautii has been shown to metabolize many flavonoid compounds from a variety of subclasses (56, 57), providing a biological basis for why one species can contribute to more than one SMC score. However, the relation of flavonoid intake with its relative abundance in fecal samples of humans is unclear, with randomized controlled trials finding no effect of polyphenol-rich diets on *F. plautii* abundance in healthy adults (58). The role of flavonoids, as distinct from other polyphenolic compounds, in determining the relative abundance of *F. plautii* requires further elucidation.

### Ecosystem-based approach to analyzing the relation of diet (flavonoid subclass intake) with the microbiome.

By adopting a bootstrap resampling approach to community identification, weighting all included microorganisms equally, and incorporating only directionality into the model, the SMC score was able to avoid carrying forward artifacts, or unintended patterns, from the original data set. We have previously shown that our profile identification method identifies a reproducible profile that outperforms studies of individual associations in its validity (35). In agreement, this current study also found more consistent associations with the profile identification method compared to individual subclass-microbe regression models. For all flavonoid subclasses, members of the SMC were consistently ranked as positive or inverse discriminators in the bootstrap resamples (Table 3). Sensitivity analyses in which the number of resamples and the random seed were varied did not have a substantial impact on results, adding further evidence to the robustness of the results.

Conversely, partial Spearman correlation models in the sample data set identified 16 associations that remained significantly associated with the respective subclass after controlling for multiple potential confounders. The consistency of the bootstrapped community results, in conjunction with the limited number of microbes that are individually associated with the subclasses, is further support that our community-based analytic ethos is appropriate. The results suggest that flavonoid subclass consumption impacts the microbiome at a community level, rather than at the level of individual species.

When conducting community-based analyses, there is often concern that community membership may be driven by an individual species, or group of species, that is both strongly associated with subclass intake and that contributes substantially to the total species abundance. To test whether this phenomenon was occurring in our analysis, we examined the correlation between all species comprising an individual SMC. As can be seen in Fig. S1 in the supplemental material, there were isolated cases of two species being cocorrelated (*P* < 0.05), but there was no observable pattern whereby many microbes were highly correlated with an individual species, nor was there any evidence of groups of microbes being highly cocorrelated with each other. As such, we concluded that community membership was not driven by a single organism, or group of organisms, but rather that each member of the community contributed to discrimination between high and low subclass consumers. This reinforces our conclusion that the association of flavonoid subclasses with the microbiome is at the community level, and not at the level of individual species.

10.1128/mBio.01205-19.1 FIG S1 Correlation between the relative abundance of species comprising each subclass-specific microbiome profile. Download FIG S1, PDF file, 0.7 MB. Copyright © 2019 Ivey et al. 2019 Ivey et al. This content is distributed under the terms of the Creative Commons Attribution 4.0 International license.

### Structure of the relationships between flavonoid subclass intake and microbiome composition.

The microbiome is an ecosystem comprised of living microorganisms interacting with the human gastrointestinal tract environment in which they reside. As such, we sought to implement a structural equation model aimed at modeling this ecosystem in the context of the nonindependence of human exposure and microbiome variables and the numerous potential, as-yet-unknown, confounders. As outlined in Table 4, despite substantial correlation between the intakes of the various flavonoid subclasses, there were less pronounced correlations between the different values of the SMC scores, which was expected given the large proportion of unique species contributing to each of the microbiome communities of each flavonoid subclass and the method utilized to identify the communities.

**TABLE 4:** Subclass-specific relations of subclass intake with subclass microbiome profile scores, after adjustment for multiple confounders and correlation of independent and dependent variable matrices^*a*^

Correlation matrix of flavonoid subclass intake^*b*^							Subclass	Estimate ± SEM	T value	*P* value	Correlation matrix of subclass-specific microbiome profile scores^*b*^						
Subclass	Flavonol	Flavanol monomer	Flavanol polymer	Flavanone	Flavone	Anthocyanidin					Subclass	Flavonol	Flavanol monomer	Flavanol polymer	Flavanone	Flavone	Anthocyanin
Flavonol	1.00						Flavonol	0.988 ± 0.495	1.995	0.046	Flavonol	1.00					
Flavanol monomer	0.69*	1.00					Flavanol monomer^*c*^	0.352 ± 0.140	2.512	0.012	Flavanol monomer	0.12	1.00				
Flavanol polymer	0.76*	0.94*	1.00				Flavanol polymer^*c*^	0.295 ± 0.139	2.128	0.033	Flavanol polymer	0.03	0.36*	1.00			
Flavanone	0.17*	0.15*	0.21*	1.00			Flavanone	0.581 ± 0.104	5.611	<0.0001	Flavanone	0.03	0.22*	0.05	1.00		
Flavone	0.45*	0.28*	0.34*	0.52*	1.00		Flavone	0.918 ± 0.385	2.384	0.017	Flavone	0.13*	0.13*	0.08	0.34*	1.00	
Anthocyanidin	0.39*	0.38*	0.47*	0.15*	0.28*	1.00	Anthocyanidin	0.455 ± 0.166	2.738	0.006	Anthocyanidin	0.13*	0.17*	0.30*	0.23*	0.44*	1.00

a Results are multivariate adjusted for the following variables: age at time of fecal collection; energy expended in physical activity; body mass index; and intakes of yogurt, calories, protein, saturated fat, trans fat, carbohydrate, and fiber. Root mean square error of approximation (RMSEA) estimate, <0.0001; Bentler comparative fit index, 1.000; Bentler-Bonett normed fit index (NFI), 0.971. A total of 247 participants were examined in this study.

b Pearson correlation coefficient. *, *P* < 0.05.

c Represents a single latent construct in the regression analysis.

The effect of flavonoids on the microbiome is not unidirectional. In fact, compounds from many flavonoid subclasses have been shown to elicit antimicrobial effects on particular microorganisms (31). As such, in addition to incorporating a set of “positive” species (that were in higher relative abundances in high subclass consumers, and lower relative abundance in low flavonoid consumers), the SMC scores also incorporated a set of “inverse” species (that were in lower relative abundances in high flavonoid consumers, and higher relative abundance in low flavonoid consumers). Given the way in which the SMC scores were computed (equation 1), it was our prespecified hypothesis that, compared to low consumers, high subclass consumers would have more of the “positive” microorganisms and fewer of the “inverse” microorganisms. As such, for each subclass, we hypothesized that subclass intake will be positively associated with the value of the corresponding microbiome community score.

The relative importance of diet and lifestyle factors in influencing diet-microbiome associations is poorly understood. When exploring the contribution of individual variables in the multivariate-adjusted model, none of the variables significantly contributed to the model (Table S2). Therefore, in addition to concluding that cocorrelation with other flavonoid subclasses was not a driver of the subclass-specific associations, we were also able to conclude that none of the included covariates significantly altered the diet-microbiome relationship in this analysis. Furthermore, the inclusion of acid-lowering medications and antibiotic medications to the multivariate-adjusted model did not substantially alter results (data not shown).

10.1128/mBio.01205-19.3 TABLE S2 Contribution of variables from the multivariate-adjusted model to subclass-specific association tests. Download Table S2, DOCX file, 0.03 MB. Copyright © 2019 Ivey et al. 2019 Ivey et al. This content is distributed under the terms of the Creative Commons Attribution 4.0 International license.

Even after adjusting for potential confounders and removing the variability that is accounted for by significant covariance with other variables in the flavonoid intake and SMC score data matrices, the intakes of all subclasses remained significantly and positively associated with the values of their respective SMC scores. These results indicate that the SMC scores we identified were indeed reflective of the intake of their respective subclass, and were not simply a marker for flavonoid intake in general, or a diet/lifestyle that is characteristic of high flavonoid consumers.

Although we could not account for unmeasured confounders of the subclass-community association, our structural equation modeling approach did enable us to draw conclusions regarding how well our model reflects the observed data. With the Bentler comparative fit and Bentler-Bonett normed fit (NF) all greater than 0.9, we conclude that the model specifying that the subclasses are the major determinants of their respective communities fits the observed data well and is an appropriate biological model for this cohort.

### Relation of whole-food intake with microbiome community scores.

To test the robustness of our identified communities and ensure translatability of results, we then sought to explore how the SMC scores were related to the wholefoods contributing most to the intakes of the corresponding subclasses outlined in Table 2. Echoing the highly significant associations observed in the subclass analyses, we observed a strong association of blueberry intake with the value of the anthocyanidin microbiome community score (Table 5). In fact, even after accounting for diet and lifestyle factors that may explain the relationship, blueberry consumption accounted for 14.2% of the variance in the community of 20 species that comprised the anthocyanidin microbiome community score (*P* < 0.0001). To ensure that this association was due to blueberries themselves, and not because blueberries may serve as a proxy for total anthocyanidin intake, we further adjusted the multivariate-adjusted model for intakes of the other whole foods contributing most to anthocyanidin intake, and the significant association remained: *R*^2^ = 14.4% and *P* < 0.0001. The anthocyanidin microbiome community score was characterized by having a lower relative abundance of Clostridium bolteae (NCBI:txid208479), which has been previously been shown to be associated with clinical infection (59).

**TABLE 5:** Subclass-specific microbiome profile score by tertiles of whole-food consumption

Subclass-specific microbiome profile score and characteristic	Parameter value^*a*^		*R*^2^ (%)	*P* for trend^*b*^
	Low consumers	High consumers		
Flavonol microbiome profile score				
Onion consumption (frequency)	≤1/wk	≥4/wk		
No. of participants	92	87		
Age and energy adjusted	−0.092 ± 0.104	0.181 ± 0.108	1.8	0.114
Multivariate adjusted^*c*^	−0.042 ± 0.107	0.119 ± 0.111	0.8	0.378
Food and multivariate adjusted^*d*^	0.113 ± 0.115	0.204 ± 0.117	0.7	0.370
Tea consumption (frequency)	Never consume	≥1/wk		
No. of participants	138	77		
Age and energy adjusted	−0.183 ± 0.084	0.226 ± 0.113**	4.1	**0.006**
Multivariate adjusted^*c*^	−0.224 ± 0.082	0.304 ± 0.111***	6.0	**<0.0001**
Food and multivariate adjusted^*d*^	−0.236 ± 0.087	0.292 ± 0.113***	5.9	**0.001**
Apple consumption (frequency)	≤0.5/wk	≥3/wk		
No. of participants	90	102		
Age and energy adjusted	−0.161 ± 0.107	0.123 ± 0.100	1.5	0.157
Multivariate adjusted^*c*^	−0.004 ± 0.119	−0.033 ± 0.109	0.1	0.879
Food and multivariate adjusted^*d*^	0.088 ± 0.126	0.102 ± 0.114	0.0	0.996
				
Flavanol monomer microbiome profile score				
Tea consumption (frequency)	Never consume	≥1/wk		
No. of participants	138	77		
Age and energy adjusted	−0.140 ± 0.083	0.391 ± 0.111***	7.3	**<0.0001**
Multivariate adjusted^*c*^	−0.161 ± 0.082	0.478 ± 0.111***	9.8	**<0.0001**
Food and multivariate adjusted^*d*^	−0.223 ± 0.087	0.458 ± 0.112***	10.4	**<0.0001**
Blueberry consumption (frequency)	≤ 0.5/wk	≥3/wk		
No. of participants	111	89		
Age and energy adjusted	−0.073 ± 0.095	0.145 ± 0.107	1.2	0.227
Multivariate adjusted^*c*^	−0.031 ± 0.099	0.093 ± 0.113	0.4	0.632
Food and multivariate adjusted^*d*^	−0.034 ± 0.100	0.041 ± 0.118	0.4	0.579
Red wine consumption (frequency)	Never consume	≥3/wk		
No. of participants	76	105		
Age and energy adjusted	0.098 ± 0.115	0.060 ± 0.097	1.7	0.126
Multivariate adjusted^*c*^	0.127 ± 0.122	0.070 ± 0.103	2.1	0.074
Food and multivariate adjusted^*d*^	0.103 ± 0.135	0.042 ± 0.103	2.3	**0.040**
				
Flavanol polymer microbiome profile score				
Tea consumption (frequency)	Never consume	≥1/wk		
No. of participants	138	77		
Age and energy adjusted	−0.119 ± 0.084	0.237 ± 0.113*	2.6	0.040
Multivariate adjusted^*c*^	−0.115 ± 0.085	0.292 ± 0.115**	3.3	0.016
Food and multivariate adjusted^*d*^	−0.088 ± 0.090	0.321 ± 0.113**	3.3	0.012
Apple consumption (frequency)	≤0.5/wk	≥3/wk		
No. of participants	90	102		
Age and energy adjusted	−0.247 ± 0.105	0.110 ± 0.098*	3.4	0.014
Multivariate adjusted^*c*^	−0.206 ± 0.119	0.076 ± 0.109	2.6	0.040
Food and multivariate adjusted^*d*^	−0.179 ± 0.127	0.093 ± 0.114	2.4	0.042
Blueberry consumption (frequency)	≤0.5/wk	≥3/wk		
No. of participants	111	89		
Age and energy adjusted	−0.227 ± 0.092	0.318 ± 0.103***	6.0	0.001
Multivariate adjusted^*c*^	−0.227 ± 0.097	0.331 ± 0.110***	5.1	0.002
Food and multivariate adjusted^*d*^	−0.172 ± 0.102	0.384 ± 0.118***	5.2	0.001
				
Flavanone microbiome profile score				
Orange consumption (frequency)	≤0.5/wk	≥3/wk		
No. of participants	109	92		
Age and energy adjusted	−0.235 ± 0.092	0.363 ± 0.102***	7.5	**<0.0001**
Multivariate adjusted^*c*^	−0.221 ± 0.096	0.376 ± 0.104***	6.9	**<0.0001**
Food and multivariate adjusted^*d*^	−0.237 ± 0.100	0.338 ± 0.108***	6.5	**<0.0001**
Red wine consumption (frequency)	Never consume	≥3/wk		
No. of participants	76	105		
Age and energy adjusted	−0.152 ± 0.113	0.185 ± 0.096*	2.7	**0.035**
Multivariate adjusted^*c*^	−0.135 ± 0.121	0.183 ± 0.102	1.8	0.103
Food and multivariate adjusted^*d*^	−0.161 ± 0.127	0.144 ± 0.101	1.5	0.137
Grapefruit consumption (frequency)	Never consume	≥0.5/wk		
No. of participants	160	87		
Age and energy adjusted	−0.001 ± 0.079	−0.009 ± 0.107	0.0	0.954
Multivariate adjusted^*c*^	0.018 ± 0.079	0.000 ± 0.108	0.0	0.897
Food and multivariate adjusted^*d*^	−0.017 ± 0.083	−0.053 ± 0.108	0.0	0.789
				
Flavone microbiome profile score				
Vegetable juice consumption (frequency)	Never consume	≥0.5/wk		
No. of participants	157	90		
Age and energy adjusted	−0.113 ± 0.079	0.188 ± 0.106*	1.9	**0.026**
Multivariate adjusted^*c*^	−0.100 ± 0.080	0.184 ± 0.109*	1.6	**0.043**
Food and multivariate adjusted^*d*^	−0.113 ± 0.080	0.210 ± 0.105*	2.0	**0.015**
Red wine consumption (frequency)	Never consume	≥3/wk		
No. of participants	76	105		
Age and energy adjusted	−0.128 ± 0.108	0.310 ± 0.092**	8.1	**<0.0001**
Multivariate adjusted^*c*^	−0.138 ± 0.116	0.328 ± 0.098**	7.5	**<0.0001**
Food and multivariate adjusted^*d*^	0.038 ± 0.121	0.402 ± 0.097*	7.2	**<0.0001**
Orange consumption (frequency)	≤0.5/wk	≥3/wk		
No. of participants	109	92		
Age and energy adjusted	−0.293 ± 0.092	0.219 ± 0.101***	6.4	**<0.0001**
Multivariate adjusted^*c*^	−0.277 ± 0.096	0.200 ± 0.104**	5.2	**0.001**
Food and multivariate adjusted^*d*^	−0.284 ± 0.093	0.181 ± 0.104**	5.0	**0.001**
				
Anthocyanidin microbiome profile score				
Blueberry consumption (frequency)	≤0.5/wk	≥3/wk		
No. of participants	111	89		
Age and energy adjusted	−0.410 ± 0.087	0.499 ± 0.098***	16.3	<0.0001
Multivariate adjusted^*c*^	−0.426 ± 0.091	0.508 ± 0.104***	14.2	<0.0001
Food and multivariate adjusted^*d*^	−0.431 ± 0.103	0.585 ± 0.109***	14.4	<0.0001
Strawberry consumption (frequency)	Never consume	≥3/wk		
No. of participants	48	52		
Age and energy adjusted	0.148 ± 0.144	0.278 ± 0.139	3.4	0.015
Multivariate adjusted^*c*^	0.185 ± 0.146	0.183 ± 0.149	2.1	0.070
Food and multivariate adjusted^*d*^	0.378 ± 0.143	−0.132 ± 0.151*	2.5	0.023
Apple consumption (frequency)	≤0.5/wk	≥3/wk		
No. of participants	90	102		
Age and energy adjusted	−0.217 ± 0.105	0.241 ± 0.099**	4.1	0.006
Multivariate adjusted^*c*^	−0.145 ± 0.119	0.143 ± 0.110	1.0	0.270
Food and multivariate adjusted^*d*^	−0.058 ± 0.120	0.198 ± 0.105	0.8	0.301

a Results are least-squared means ± standard errors of the means (SEMs) by ANCOVA. Values that are significantly different from the values for low consumers are indicated by asterisks as follows: *, *P* < 0.05; **, *P* < 0.01; **, *P* < 0.001; ***, *P* < 0.0001. A total of 247 participants were examined in this study.

b *P* values for trend. Significant values are shown in boldface type.

c The multivariate-adjusted model includes the following variables: age at time of fecal collection; energy expended in physical activity; body mass index; and intakes of yogurt, calories, protein, saturated fat, trans fat, carbohydrate, and fiber.

d Includes adjustment for variables in the multivariate-adjusted model as well as adjustment for the other subclass-specific whole foods.

A similar strong association was observed for tea intake, where even after adjusting for multiple potential confounders and the consumption of other flavonol-rich foods, tea consumption explained 5.9% of the unweighted variance in the 20 species comprising the flavonol microbiome community score (*P* = 0.001). The flavonol microbiome community was characterized by having lower relative abundance of Faecalibacterium prausnitzii (NCBI:txid853) which has been implicated as having beneficial effects on human health (60), and a greater relative abundance of the flavonoid-metabolizing bacterium *E. lenta*, which has been associated with frailty in the elderly (61) and inactivates the cardiac drug digoxin (62). It is unclear how the health benefits of flavonol intake interact, as shown by an abundant literature of the benefit of tea consumption (63), with the nonbeneficial features of the community of bacteria associated with high flavonol intake, as well as the other community members where their importance to human health is not yet clearly elucidated.

The percent contribution whole foods make to total intake of a given subclass did not appear to drive the strength of associations. In fact, for the flavanol polymer subclass, although all three major whole-food contributors were significantly associated with the value of the flavanol polymer microbiome community score, the top contributor, tea, accounting for 28.1% of total flavanol polymer intake, explained only 2.6% of the flavone microbiome community score variance. Conversely, 6.0% of total variance in the species comprising the flavanol polymer microbiome community was explained by the consumption of blueberries, which contributed only 11.4% of total dietary flavanol polymers. The association of flavanol polymer-rich foods independent from other flavanol polymer-rich foods highlights the potential effect that flavanol polymer-rich foods, generally, have on the community of 20 microorganisms that collectively make up the flavonol polymer microbiome community score.

Due to the observational nature of this study, as well as the measurement error associated with assessing flavonoid intake and microbiome composition, there is an increased likelihood for type two statistical errors to occur, tending results toward the null hypothesis of no association. Measurement error can also contribute to an underestimation of true effects, resulting in effect estimates that are smaller than what truly occurs in the population. On the other hand, there is the chance that the lack of an external validation cohort may contribute to an overestimation of the true magnitude of effect. However, indications of specificity for certain foods, percent contribution of whole foods to subclass intake not driving results, and significant results remaining even after adjusting for other major whole-food subclass contributors, all point toward a lack of bias and an absence of overestimation. Having said this, future studies validating this model in a variety of populations will be useful in establishing population-specific magnitude of effects.

### Conclusion.

Using an ecosystem-based model, we identified six microbial communities uniquely and independently associated with intakes of the six flavonoid subclasses, and the main foods that contribute to their intake. Strengthening our understanding of how diet alters the microbiome, we found that in this cohort of healthy adult males, flavonoids were associated with microbial community assembly, independent from effects of other dietary and lifestyle factors included in the multivariate-adjusted model. In other words, the importance of the presented subclass-specific microbial communities (SMCs) is that there is a pool of bacteria that variously assimilate flavonoids, use by-products from assimilating bacteria, or are members of an impacted ecological niche, that are notably different from other consumers and nonconsumers of flavonoids. The driver of the ecological organization (a flavonoid compound ingested) results in SMCs that are detected. By confirming the role of diet in influencing microbiome structure in a population community-based setting, within a context of numerous potential dietary and lifestyle confounders, we build upon seminal work by David et al. (32), who established the role of diet in shaping the composition of the microbiome in intervention studies.

In this cohort of generally healthy U.S. males, intakes of flavonoid-rich foods explained a large proportion of total community variance. In fact, consuming tea at least once per week explained 10.4% of the total variance in assembly of the 20 species comprising the flavanol monomer microbial community score. The novel methodology employed, necessitated by multidimensional microbiome data that consist of nonindependent features that exhibit a wide range of distributions and mean values, has considerable value beyond the presented work and addresses a major challenge in our ability to understand associations of the microbiome in a wide range of clinical and epidemiologic settings. Further work in this area is indicated to fully elucidate the relation identified microorganisms have with human health.

## MATERIALS AND METHODS

### Population.

The Men’s Lifestyle Validation Study (64, 65) was a 1-year substudy of the Health Professionals Follow-up Study (66, 67) which included 51,529 U.S. health professionals whose diet, lifestyle, and health was monitored every 2 years from 1986 to present day. Diet was assessed by a validated semiquantitative food frequency questionnaire (SFFQ) every 4 years. Study protocol 22067–102 titled “Men’s Lifestyle Validation Study and Microbiome Correlation” was approved by the Harvard T. H. Chan School of Public Health Institutional Review Board, and informed consent was obtained from all participants.

In 2012, we randomly selected a subset (247 included in analysis) of Health Professionals Follow-up Study participants aged 45 to 80 years, from all geographical regions of the United States, who had completed the 2006/2007 cohort SFFQ, had previously provided blood samples, and had access to the Internet. Men with a history of coronary heart disease, stroke, cancer, or major neurological disease were excluded, as were participants with SFFQ total daily energy intakes of <600 kcal or >3,500 kcal, or with more than 70 blank SFFQ items. Over the 1-year duration of the Men’s Lifestyle Validation Study, participants provided up to four stool samples (64). The third fecal sample, collected approximately 6 months into the study, was used in all analyses presented in this paper, as it yielded the greatest number of participants with complete fecal sample and dietary assessment for inclusion in this study (247 out of 308). Methods for fecal sample collection, nucleic acid extraction, and taxonomic profiling have been previously described (64, 65).

### Flavonoid intake assessment.

Participants completed a SFFQ to indicate habitual dietary intake over the year preceding sample collection. From this, habitual daily intake, in milligrams/day, of total flavonoids and flavonoid subclasses was estimated using previously described methods (9, 68). Flavonoid subclasses in this analysis include the following: (i) flavonols; (ii) flavanol monomers (including catechins and epicatechins and excluding proanthocyanins); (iii) flavanol polymers (including proanthocyanins, theaflavins, and thearubigins); (iv) flavones; (v) flavanones; and (vi) anthocyanins. Frequency of consumption of flavonoid-rich foods were recorded as the number of servings per day, week, or month (68).

### Covariate assessment.

At enrollment, participants reported date of birth, height, geographic location, ethnicity, and smoking status. Other measures, such as Bristol stool score, and the use of acid-lowering and antibiotic medications, were collected via questionnaire at the time of fecal sample collection. Weight and height at enrollment were used to calculate body mass index (BMI) (in kilograms/square meter), calculated as weight divided by the square of height.

### Identification of flavonoid-metabolizing bacteria.

Limiting the search to bacteria, we searched the BRENDA database (47) for enzymes with substrates containing the text substrings outlined in Appendix A. A dietitian then curated the results to include only those reactions involving dietary flavonoid compounds.

### Statistical analysis. (i) Creation of the subclass-specific microbial community scores.

Alpha diversity, membership, was assessed using the Shannon H’ index. We removed all taxonomic features with a relative abundance of less than 10^−4^ (0.01%) in greater than 10% of all samples. After applying this statistical filter, 140 species remained and were included in the subsequent subclass-specific canonical discriminant analyses. The species data were normalized via arcsine square root transformation. To ensure that all species had the opportunity to contribute equally to the canonical models, the values were also standardized across participants (mean, 0; standard deviation, ±1). For this species identification step, intakes of each of the six flavonoid subclasses were divided into three groups based on the level of subclass consumption; low consumers (quintiles 1 and 2 of subclass intake), moderate consumers (quintile 3), and high consumers (quintiles 4 and 5 of subclass intake).

To identify the subclass-specific microbial communities (SMCs), the following analyses were conducted separately, once for each flavonoid subclass. To reduce the impact of data artifacts influencing which species were included in the SMCs, the cohort data were bootstrapped with 500 resamples. For each bootstrap resample, species were omitted from the analysis if either total or within-group variance equaled zero. In each bootstrap resample, to identify the pattern of species that differentiated low from high subclass consumers, we implemented a canonical discriminant analysis model of subclass intake (low versus high, excluding moderate consumers) against all detected species, without any knowledge of intakes of other flavonoid subclasses or potentially confounding variables. Within each bootstrap resample, species were then ranked by their positive and inverse discriminatory capacity using the canonical coefficients.

For each flavonoid subclass, we then pooled data from each bootstrap resample and summed the species rankings. In order to identify the species that, when considered as a community, are present in greater relative abundance in high subclass consumers compared to low subclass consumers, we identified positive discriminators as defined as the species in the top ten of pooled positive discriminatory rankings of the canonical coefficients. Conversely, the species that, as a whole, are present in lower relative abundance in high subclass consumers than low consumers, are defined as the inverse discriminators and represent the species in the top ten of pooled inverse discriminatory rankings of the canonical coefficients. These positive and inverse discriminators were then used to calculate the SMC scores using the method outlined in equation 1 (35). The subclass-specific microbiome profile score was computed as follows:

(1) $$\text{microbiome profile score}=\frac{1}{n}\overset{n=10}{\underset{i=1}{\sum }}h_{i}-\frac{1}{n}\overset{n=10}{\underset{i=1}{\sum }}l_{i}$$

where *h* is the relative abundance of species with the greatest pooled positive discriminatory rankings and *l* is the relative abundance of species with the greatest pooled inverse discriminatory rankings. The value of ten species was selected as we have previously shown this value to provide superior discriminatory capacity and validity to other manifestations of equation 1. We repeated this analysis, altering the number of bootstrap resamples, and results were not dramatically altered. To enable comparisons across subclasses, each SMC score was standardized to a mean of 0 and a standard deviation of 1. As a sensitivity analysis, we repeated the bootstrap resamples by changing the number of resamples and the value of the seed.

To test the association of individual microbes comprising the communities, we conducted partial Spearman rank correlation analyses. In line with variables differing between high and low total-flavonoid consumers outlined in Table 1, this model corrected for the following: age at time of fecal collection; energy expended in physical activity; body mass index; and intake of yogurt, total calories, protein, saturated fat, trans fat, carbohydrate, and fiber.

**(ii) Structure of relations between subclass intake and subclass-specific microbial community scores.** We then implemented a structural equation model that simultaneously regressed subclass intake against its corresponding SMC score (in a multivariate-adjusted model), while concurrently adjusting for any significant cocorrelation in the dependent and independent variable matrices. Intakes of flavanol monomers and flavanol polymers were highly correlated (Pearson correlation coefficient = 0.94). As such, a monomer/polymer latent variable was constructed to represent the shared covariance in intakes of flavanol monomers and flavanol polymers. This latent construct was then used as the independent variable for the flavanol monomer and flavanol polymer subclass-specific association tests.

To observe the proportion of variance in the SMC score that is directly attributable to intake of its corresponding flavonoid subclass, we presented results as the B estimate ± standard error, T value, and *P* value.

**(iii) Relation of whole-food intake with subclass-specific microbial communities.** As a means of examining the robustness and translatability of the SMC scores, for each flavonoid subclass, we identified the three major whole-food contributors to subclass intake. Frequency of consumption of each of the whole foods was first trichotomized into three groups based on tertiles of consumption frequency. We then implemented age- and energy-adjusted and multivariate-adjusted analyses of covariance (ANCOVA) models to test whether the value of the SMC score differed across tertiles of whole-food intake.

To observe the proportion of variance in the SMC that is directly attributable to intake of its corresponding flavonoid subclass, we presented results as the mean (± standard error of the mean) of the SMC score, the partial *R*^2^ (coefficient of partial determination), and the *P* value.

All analyses were performed with SAS 9.2 statistical package.

### Data availability.

Sequence data have been deposited in the Sequence Read Archive under BioProject ID PRJNA354235. Data from the Health Professionals Follow-up Study (HPFS), including metadata not included in the current manuscript but collected as a part of the Men’s Lifestyle Validation Study (MLVS), can be obtained through written application. As per standard controlled-access procedure, applications to use HPFS resources will be reviewed by our External Collaborators Committee for scientific aims, evaluation of the fit of the data for the proposed methodology and verification that the proposed use meets the guidelines of the Ethics and Governance Framework and the consent that was provided by the participants. Investigators wishing to use HFPS or MLVS cohort data are asked to submit a brief (two pages) description of the proposed project (“letter of intent”) to E. B. Rimm (erimm@hsph.harvard.edu).

## APPENDIX A

**Text substrings utilized in the BRENDA database search.** The following text substrings were utilized in our prespecified BRENDA database search strategy: cyanidin; flavylium; ideain; kuromanin; keracyanin; delphinidin; malvidin; malvin; pelargonidin; peonidin; petunidin; pigment; pinotin; vitisin; butein; chalcone; phloretin; phloridzin; myricetin; quercetin; astilbin; catechin; flavanone; flavane; taxifolin; distylin; cinnamtannin; tetramer d; flavin; bonannione; mimulone; naringenin; flavanone; bavachin; didymin; eriodictyol; hesperetin; hesperidin; xanthohumol; prunin; narirutin; eriocitrin; chrysin; pinocembrin; poncirin; sakuranetin; flavone; luteolin; citrifolioside; apigenin; vicenin; vitexin; apiin; baicalein; chrysoeriol; eupatorin; diosmin; cirsimaritin; cirsilineol; scutellarein; skrofullein; diosmetin; gardenin; geraldone; hispidulin; rhoifolin; jaceosidin; orientin; scolymoside; nepetin; nobiletin; pebrellin; rhoifolin; tangeretin; scutellarein; sinensetin; kaempferol; galangin; rhamnetin; jaceidin; kaempferide; trifolin; astragalin; nicotiflorine; morin; patuletin; avicularin; hyperoside; hyperin; quercitrin; quercetrin; rutin; reynoutrin; spiraeoside; spinacetin; genisteol; neochanin; pratol; daidzin; genistin; glycitin; genistein; pratensol; biochanin; daidzein; daidzeol; formononetin; prunetol; sophoricol; glycitein; flavonoid; flavonol; flavanol; anthocyan.
