# Supplementary material for: Role of Dietary Flavonoid Compounds in Driving Patterns of Microbial Community Assembly
Source: mBio. 2019 Sep 24;10(5):e01205-19. doi: 10.1128/mBio.01205-19 (PMC6759757; doi:10.1128/mBio.01205-19)
Supplement: TABLE S1 [file mBio.01205-19-st001.docx]

**Supplemental Table 1:** Flavonoid metabolizing bacterial species identified in the BRENDA database, with the associated co-substrate (blue panel).
