# Supplementary material for: Role of Dietary Flavonoid Compounds in Driving Patterns of Microbial Community Assembly
Source: mBio. 2019 Sep 24;10(5):e01205-19. doi: 10.1128/mBio.01205-19 (PMC6759757; doi:10.1128/mBio.01205-19)
Supplement: FIG S1 [file mBio.01205-19-sf001.pdf]

a)

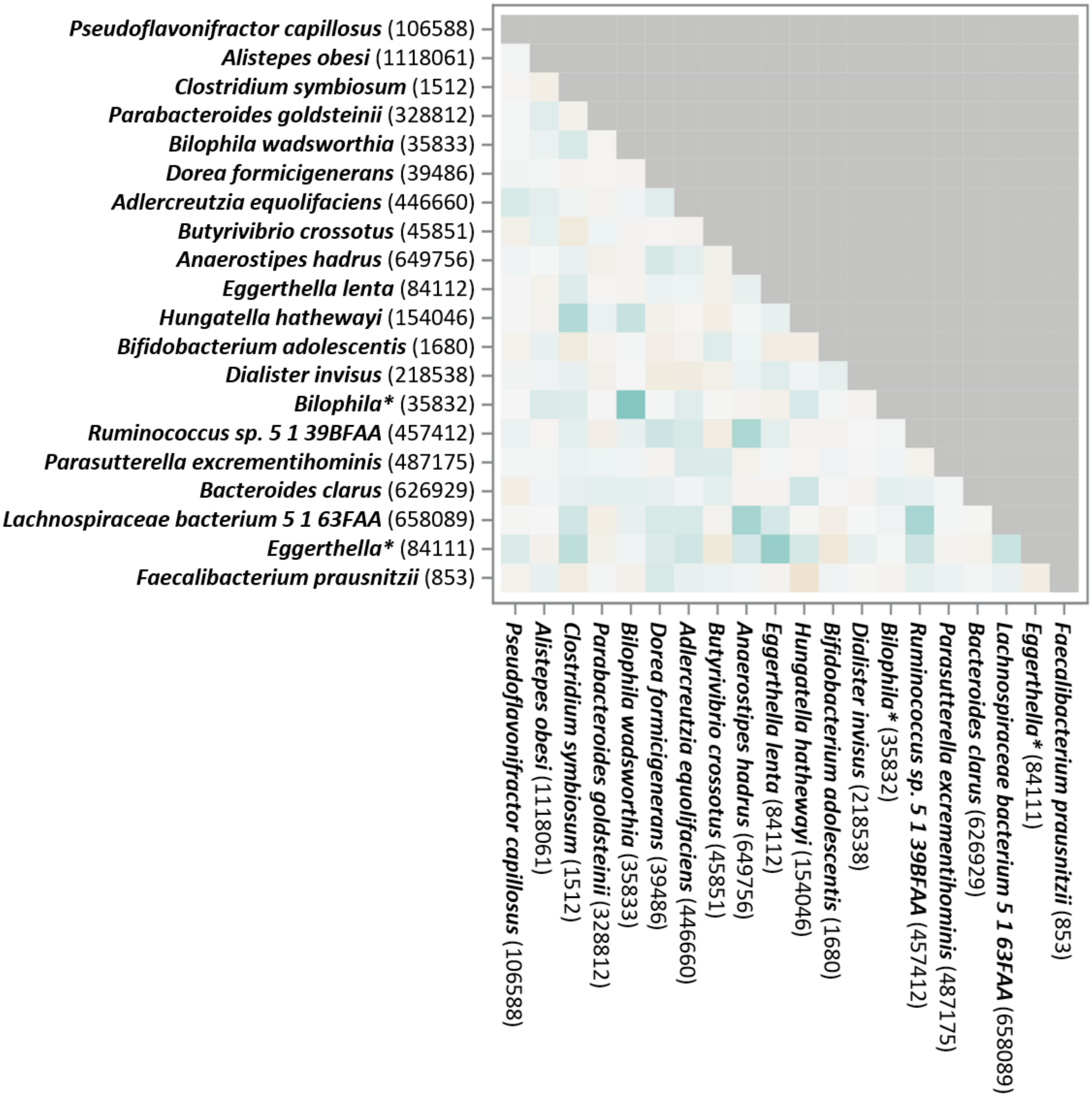

Pseudoflavonifractor capillosus (106588)
Alistepes obesi (1118061)
Clostridium symbiosum (1512)
Parabacteroides goldsteinii (328812)
Bilophila wadsworthia (35833)
Dorea formicigenerans (39486)
Adlercreutzia equolifaciens (446660)
Butyrivibrio crossotus (45851)
Anaerostipes hadrus (649756)
Eggerthella lenta (84112)
Hungatella hathewayi (154046)
Bifidobacterium adolescentis (1680)
Dialister invisus (218538)
Bilophila* (35832)
Ruminococcus sp. 5 1 39BFAA (457412)
Parasutterella excrementihominis (487175)
Bacteroides clarus (626929)
Lachnospiraceae bacterium 5 1 63FAA (658089)
Eggerthella* (84111)
Faecalibacterium prausnitzii (853)

b)

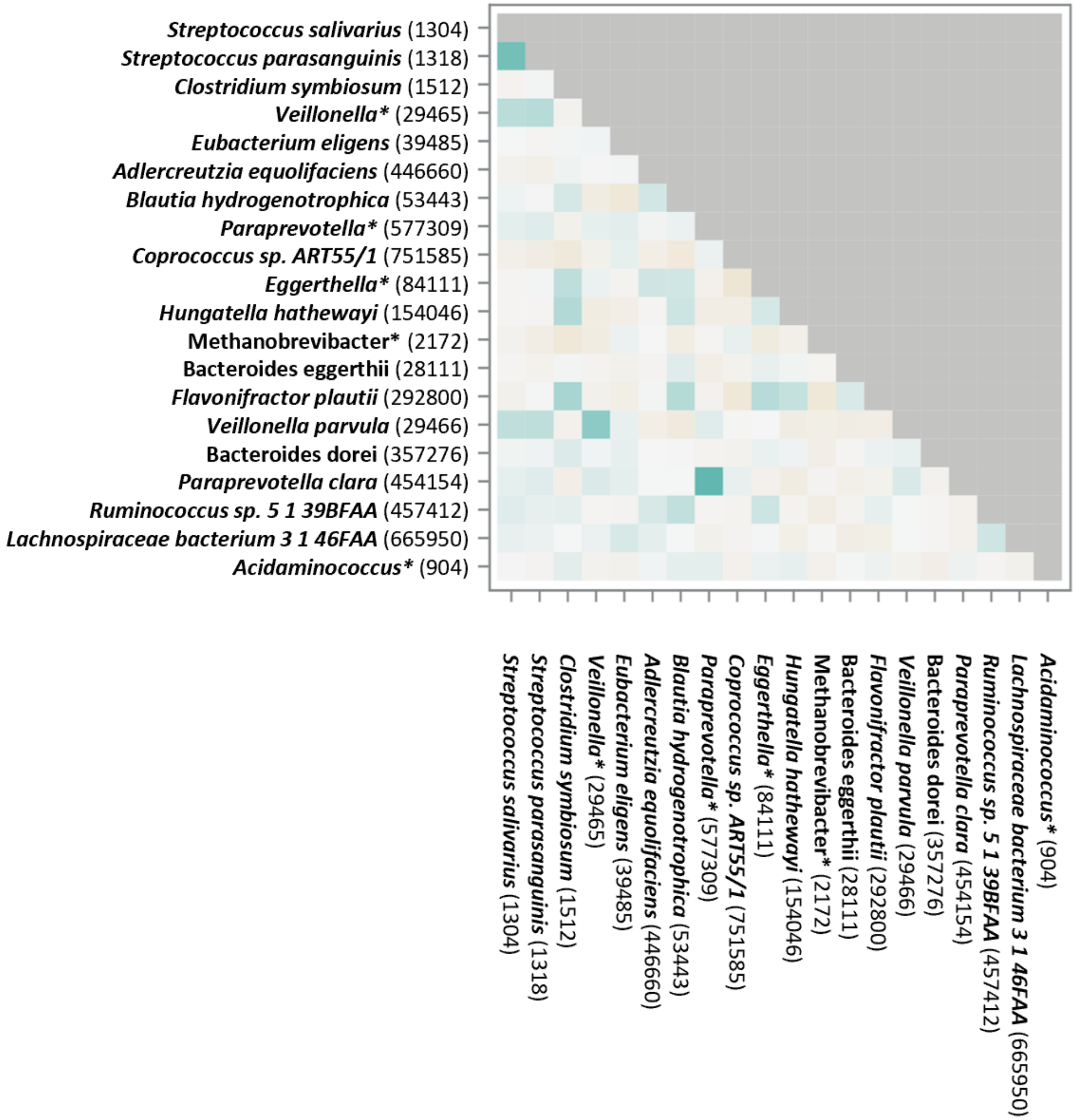
Streptococcus salivarius (1304)
Streptococcus parasanguinis (1318)
Clostridium symbiosum (1512)
Veillonella* (29465)
Eubacterium eligens (39485)
Adlercreutzia equolifaciens (446660)
Blautia hydrogenotrophica (53443)
Paraprevotella* (577309)
Coprococcus sp. ART55/1 (751585)
Eggerthella* (84111)
Hungatella hathewayi (154046)
Methanobrevibacter* (2172)
Bacteroides eggerthii (28111)
Flavonifractor plautii (292800)
Veillonella parvula (29466)
Bacteroides dorei (357276)
Paraprevotella clara (454154)
Ruminococcus sp. 5 1 39BFAA (457412)
Lachnospiraceae bacterium 3 1 46FAA (665950)
Acidaminococcus* (904)

c)

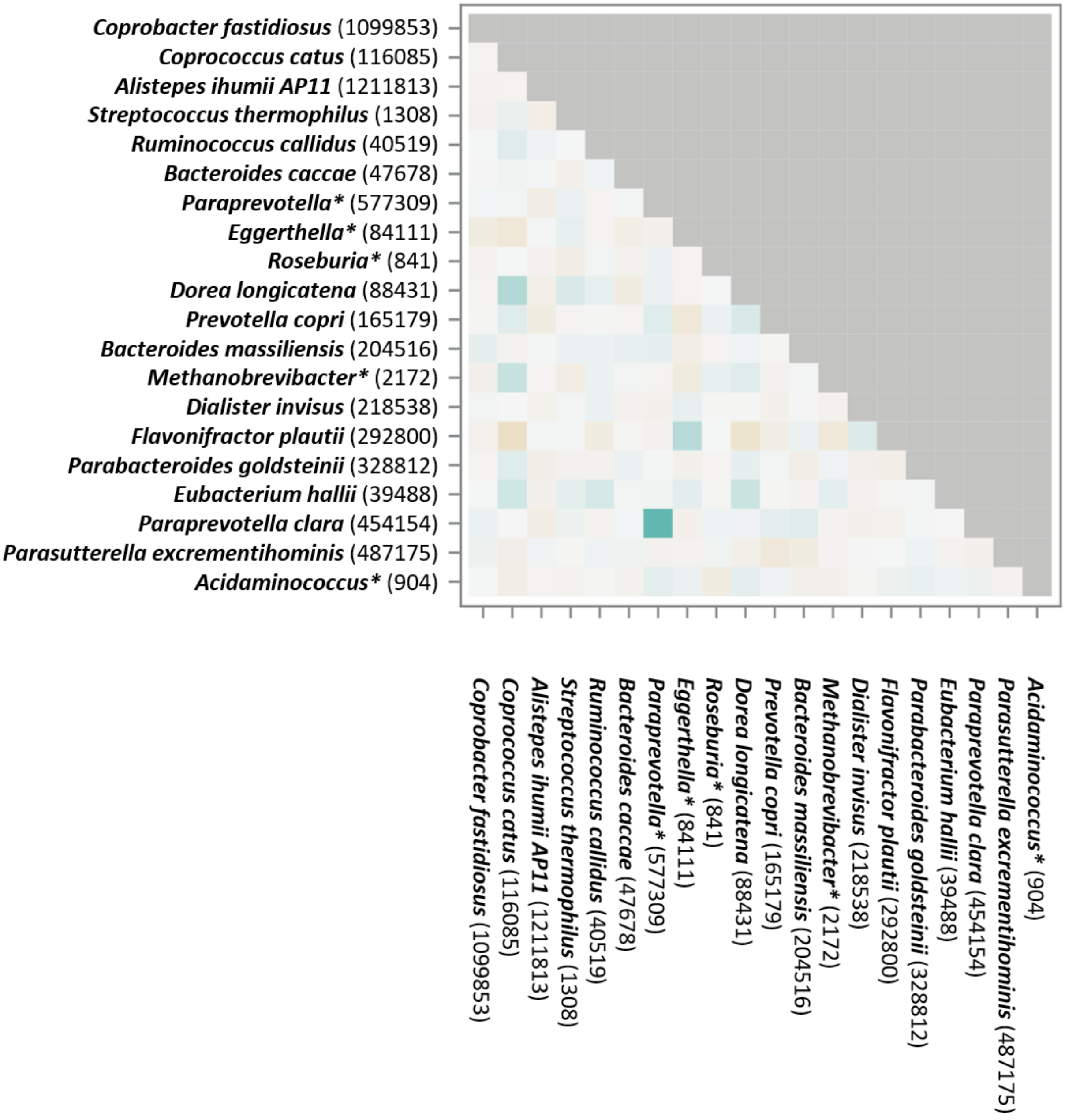

Coprobacter fastidiosus (1099853)
Coprococcus catus (116085)
Alistepes ihumii AP11 (1211813)
Streptococcus thermophilus (1308)
Ruminococcus callidus (40519)
Bacteroides caccae (47678)
Paraprevotella* (577309)
Eggerthella* (84111)
Roseburia* (841)
Dorea longicatena (88431)
Prevotella copri (165179)
Bacteroides massiliensis (204516)
Methanobrevibacter* (2172)
Dialister invisus (218538)
Flavonifractor plautii (292800)
Parabacteroides goldsteinii (328812)
Eubacterium hallii (39488)
Paraprevotella clara (454154)
Parasutterella excrementihominis (487175)
Acidaminococcus* (904)

d)

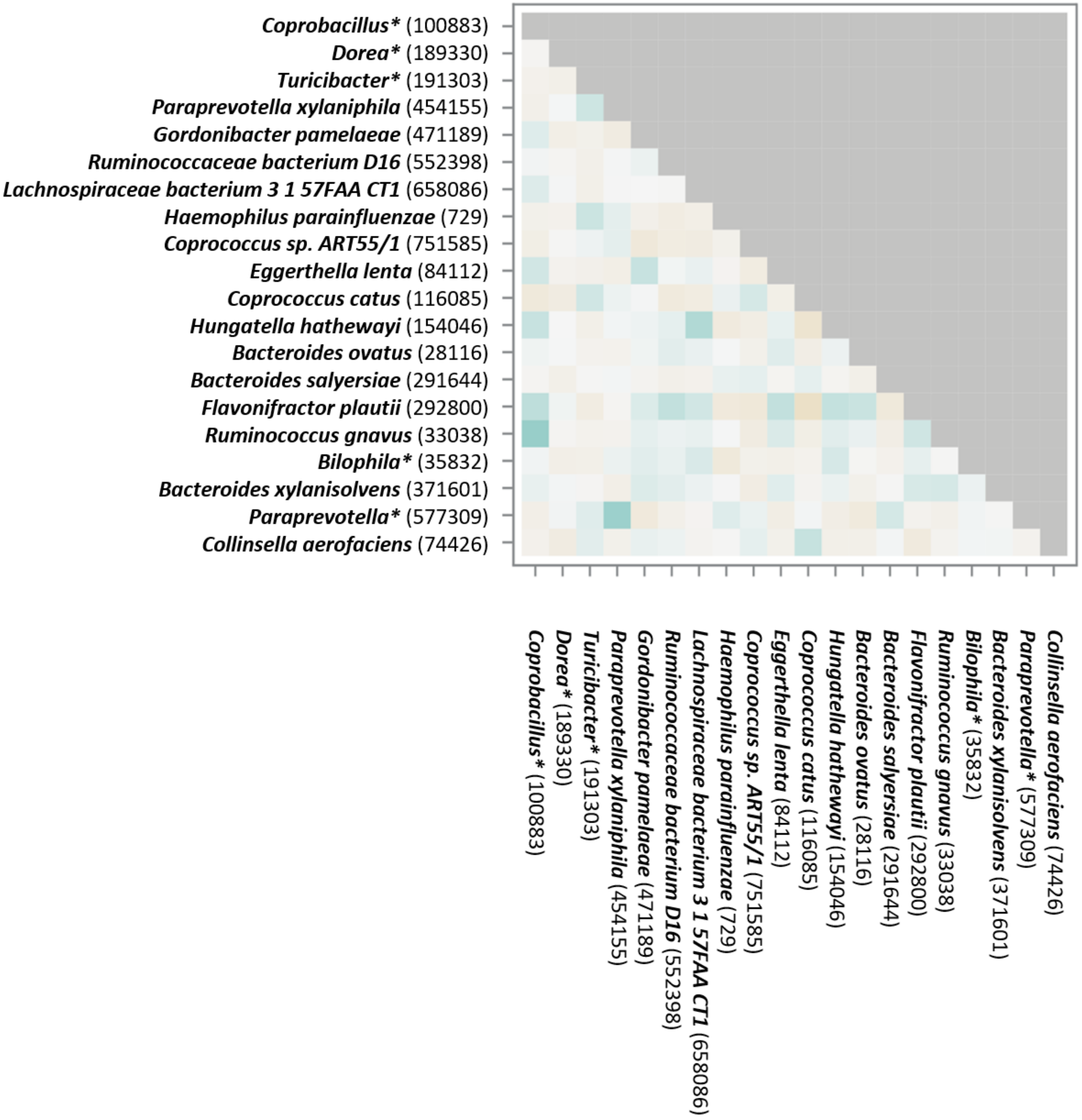

*Coprobacillus** (100883)
*Dorea** (189330)
*Turicibacter** (191303)
Paraprevotella xylaniphila (454155)
Gordonibacter pamelaeae (471189)
Ruminococcaceae bacterium D16 (552398)
Lachnospiraceae bacterium 3 1 57FAA CT1 (658086)
Haemophilus parainfluenzae (729)
Coprococcus sp. ART55/1 (751585)
Eggerthella lenta (84112)
Coprococcus catus (116085)
Hungatella hathewayi (154046)
Bacteroides ovatus (28116)
Bacteroides salyersiae (291644)
Flavonifractor plautii (292800)
Ruminococcus gnavus (33038)
*Bilophila** (35832)
Bacteroides xylanisolvens (371601)
*Paraprevotella** (577309)
Collinsella aerofaciens (74426)

e)

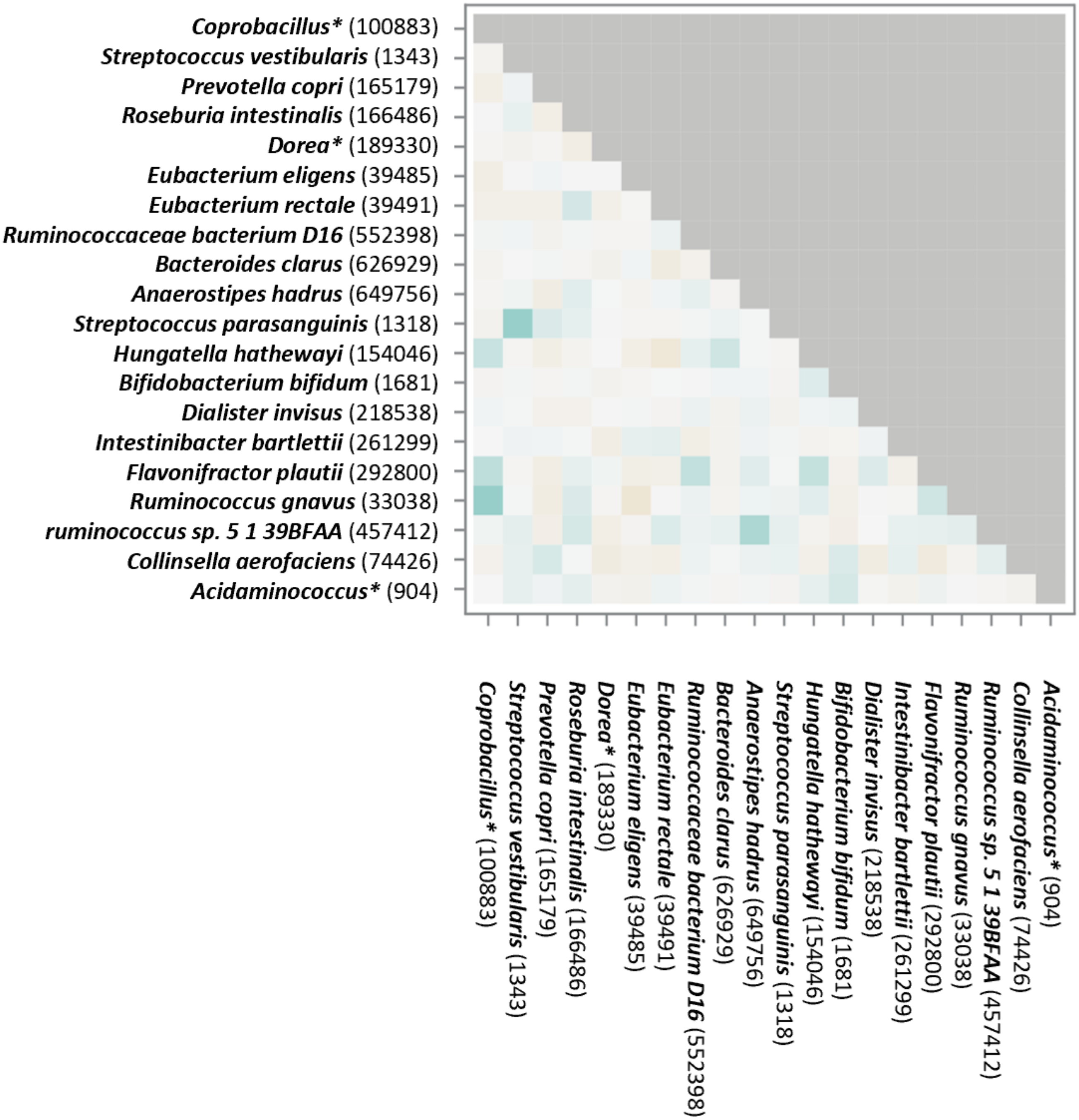
Coprobacillus* (100883)
Streptococcus vestibularis (1343)
Prevotella copri (165179)
Roseburia intestinalis (166486)
Dorea* (189330)
Eubacterium eligens (39485)
Eubacterium rectale (39491)
Ruminococcaceae bacterium D16 (552398)
Bacteroides clarus (626929)
Anaerostipes hadrus (649756)
Streptococcus parasanguinis (1318)
Hungatella hathewayi (154046)
Bifidobacterium bifidum (1681)
Dialister invisus (218538)
Intestinibacter bartlettii (261299)
Flavonifractor plautii (292800)
Ruminococcus gnavus (33038)
ruminococcus sp. 5 1 39BFAA (457412)
Collinsella aerofaciens (74426)
Acidaminococcus* (904)

f)

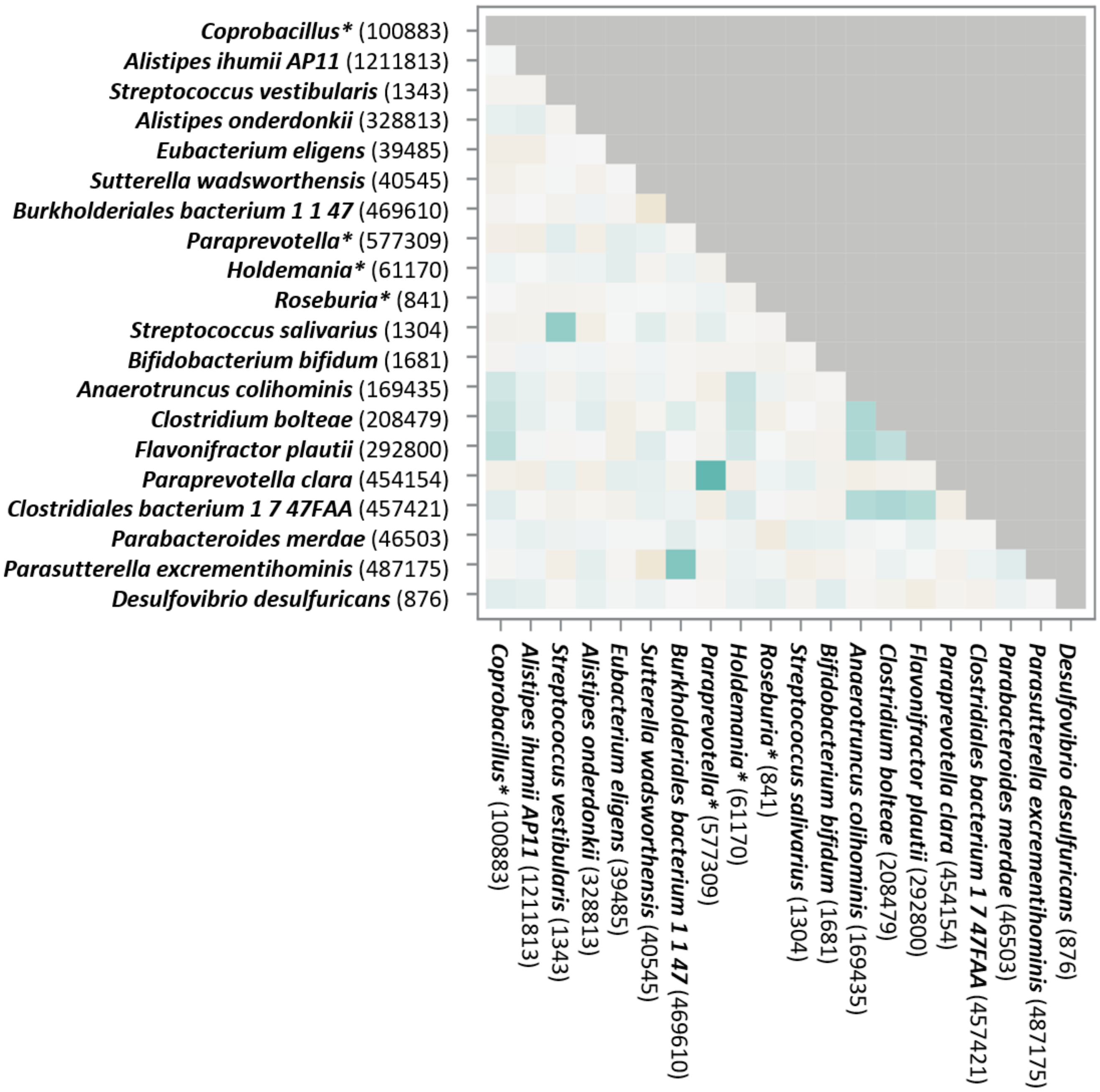

Coprobacillus* (100883)
Alistipes ihumii AP11 (1211813)
Streptococcus vestibularis (1343)
Alistipes onderdonkii (328813)
Eubacterium eligens (39485)
Sutterella wadsworthensis (40545)
Burkholderiales bacterium 1 1 47 (469610)
Paraprevotella* (577309)
Holdemania* (61170)
Roseburia* (841)
Streptococcus salivarius (1304)
Bifidobacterium bifidum (1681)
Anaerotruncus colihominis (169435)
Clostridium bolteae (208479)
Flavonifractor plautii (292800)
Paraprevotella clara (454154)
Clostridiales bacterium 1 7 47FAA (457421)
Parabacteroides merdae (46503)
Parasutterella excrementihominis (487175)
Desulfovibrio desulfuricans (876)

Supplemental Figure 1: Correlation between the relative abundance of species comprising each Subclass-specific Microbiome Profile.

2a) *Correlation matrix of species comprising the Flavonol Microbiome Fingerprint Score.*

2b) *Correlation matrix of species comprising the Flavanol Monomer Microbiome Fingerprint Score.*

2c) *Correlation matrix of species comprising the Flavanol Polymer Microbiome Fingerprint Score.*

2d) *Correlation matrix of species comprising the Flavanone Microbiome Fingerprint Score.*

2e) *Correlation matrix of species comprising the Flavone Microbiome Fingerprint Score.*

2f) *Correlation matrix of species comprising the Anthocyanidin Microbiome Fingerprint Score*

n=247.

NCBI taxonomy identification numbers presented in parenthesis.

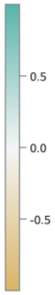

Legend: Spearman Rank correlation coefficient
