# Supplementary material for: Role of Dietary Flavonoid Compounds in Driving Patterns of Microbial Community Assembly
Source: mBio. 2019 Sep 24;10(5):e01205-19. doi: 10.1128/mBio.01205-19 (PMC6759757; doi:10.1128/mBio.01205-19)
Supplement: TABLE S2 [file mBio.01205-19-st002.docx]

| **Supplementary Table 2:** Contribution of variables from the multivariate-adjusted model to subclass-specific association tests *^a^* | | | | | | | | | | | | | | | | | |  |
| --- | --- | --- | --- | --- | --- | --- | --- | --- | --- | --- | --- | --- | --- | --- | --- | --- | --- | --- |
|  | **Flavonol** | |  | **Flavanol monomer** | |  | **Flavanol polymer** | |  | **Flavanone** | |  | **Flavone** | |  | **Anthocyanidin** | | |
|  | **Estimate**  ± SEM | **P value** |  | **Estimate**  ± SEM | **P value** |  | **Estimate**  ± SEM | **P value** |  | **Estimate**  ± SEM | **P value** |  | **Estimate**  ± SEM | **P value** |  | **Estimate**  ± SEM | **P value** | |
| **Age** *(years)* | -0.048 ± 0.148 | 0.746 |  | -0.001 ± 0.097 | 0.993 |  | 0.033 ± 0.096 | 0.734 |  | 0.044 ± 0.088 | 0.617 |  | -0.073 ± 0.141 | 0.604 |  | 0.017 ± 0.095 | 0.860 | |
| **Physical activity** *(METs)* | -0.164 ± 0.166 | 0.324 |  | -0.013 ± 0.100 | 0.893 |  | -0.046 ± 0.099 | 0.643 |  | 0.072 ± 0.090 | 0.422 |  | -0.027 ± 0.149 | 0.857 |  | 0.031 ± 0.098 | 0.753 | |
| **Body mass index** *(kg/m^2^)* | -0.117 ± 0.154 | 0.447 |  | 0.066 ± 0.101 | 0.513 |  | -0.070 ± 0.100 | 0.482 |  | -0.050 ± 0.091 | 0.587 |  | 0.173 ± 0.158 | 0.274 |  | -0.072 ± 0.099 | 0.469 | |
| **Yogurt intake** *(g/d)* | 0.111 ± 0.115 | 0.475 |  | -0.001 ± 0.104 | 0.996 |  | 0.046 ± 0.103 | 0.656 |  | 0.041 ± 0.095 | 0.664 |  | 0.116 ± 0.168 | 0.491 |  | -0.052 ± 0.104 | 0.616 | |
| **Caloric intake** *(kcal/d)* | -0.487 ± 0.330 | 0.139 |  | -0.051 ± 0.111 | 0.646 |  | 0.024 ± 0.110 | 0.831 |  | -0.077 ± 0.100 | 0.442 |  | -0.359 ± 0.306 | 0.241 |  | -0.004 ± 0.103 | 0.967 | |
| **Protein intake** *(g/d)* | -0.029 ± 0.141 | 0.840 |  | 0.045 ± 0.099 | 0.651 |  | -0.025 ± 0.099 | 0.799 |  | 0.124 ± 0.091 | 0.171 |  | 0.027 ± 0.143 | 0.849 |  | 0.056 ± 0.098 | 0.566 | |
| **Saturated fat intake** *(g/d)* | -0.040 ± 0.184 | 0.828 |  | 0.083 ± 0.130 | 0.524 |  | 0.115 ± 0.129 | 0.371 |  | 0.078 ± 0.118 | 0.510 |  | 0.224 ± 0.220 | 0.310 |  | -0.041 ± 0.129 | 0.752 | |
| **Trans fat intake** *(g/d)* | 0.262 ± 0.241 | 0.276 |  | -0.138 ± 0.130 | 0.289 |  | -0.029 ± 0.129 | 0.822 |  | -0.079 ± 0.114 | 0.487 |  | 0.043 ± 0.190 | 0.823 |  | 0.128 ± 0.142 | 0.367 | |
| **Carbohydrate intake** *(g/d)* | 0.056 ± 0.198 | 0.777 |  | 0.007 ± 0.108 | 0.946 |  | 0.051 ± 0.107 | 0.633 |  | -0.198 ± 0.101 | 0.051 |  | 0.184 ± 0.202 | 0.362 |  | 0.012 ± 0.109 | 0.913 | |
| **Fiber intake** *(g/d)* | -0.230 ± 0.297 | 0.439 |  | 0.027 ± 0.118 | 0.816 |  | 0.061 ± 0.117 | 0.601 |  | 0.067 ± 0.105 | 0.528 |  | -0.005 ± 0.197 | 0.979 |  | 0.037 ± 0.133 | 0.783 | |
| *^a^ Results are mutually adjusted. n=247* | | | | | | | | | | | | | | | | | | |
